# Integrated analysis and validation of ferroptosis-related genes and immune infiltration in acute myocardial infarction

**DOI:** 10.1186/s12872-023-03622-z

**Published:** 2024-02-24

**Authors:** Xinyu Wu, Jingru Li, Shengjie Chai, Chaguo Li, Si Lu, Suli Bao, Shuai Yu, Hao Guo, Jie He, Yunzhu Peng, Huang Sun, Luqiao Wang

**Affiliations:** 1 Department of Cardiology, The First Affiliated Hospital of Kunming Medical University, Kunming, China; 2 Department of Nephrology, The First Affiliated Hospital of Kunming Medical University, Kunming, China

**Keywords:** Ferroptosis, Acute myocardial infarction, Biomarker, CeRNA network, Immune infiltration

## Abstract

**Background:**

Acute myocardial infarction (AMI) is indeed a significant cause of mortality and morbidity in individuals with coronary heart disease. Ferroptosis, an iron-dependent cell death, is characterized by the accumulation of intracellular lipid peroxides, which is implicated in cardiomyocyte injury. This study aims to identify biomarkers that are indicative of ferroptosis in the context of AMI, and to examine their potential roles in immune infiltration.

**Methods:**

Firstly, the GSE59867 dataset was used to identify differentially expressed ferroptosis-related genes (DE-FRGs) in AMI. We then performed gene ontology (GO) and functional enrichment analysis on these DE-FRGs. Secondly, we analyzed the GSE76591 dataset and used bioinformatic methods to build ceRNA networks. Thirdly, we identified hub genes in protein–protein interaction (PPI) network. After obtaining the key DE-FRGs through the junction of hub genes with ceRNA and least absolute shrinkage and selection operator (LASSO). ImmucellAI was applied to estimate the immune cell infiltration in each sample and examine the relationship between key DE-FRGs and 24 immunocyte subsets. The diagnostic performance of these genes was further evaluated using the receiver operating characteristic (ROC) curve analysis. Ultimately, we identified an immune-related ceRNA regulatory axis linked to ferroptosis in AMI.

**Results:**

Among 56 DE-FRGs identified in AMI, 41 of them were integrated into the construction of competitive endogenous RNA (ceRNA) networks. TLR4 and PIK3CA were identified as key DE-FRGs and PIK3CA was confirmed as a diagnostic biomarker for AMI. Moreover, CD4_native cells, nTreg cells, Th2 cells, Th17 cells, central-memory cells, effector-memory cells, and CD8_T cells had higher infiltrates in AMI samples compared to control samples. In contrast, exhausted cells, iTreg cells, and Tfh cells had lower infiltrates in AMI samples. Spearman analysis confirmed the correlation between 24 immune cells and PIK3CA/TLR4. Ultimately, we constructed an immune-related regulatory axis involving XIST and OIP5-AS1/miR-216a/PIK3CA.

**Conclusion:**

Our comprehensive analysis has identified PIK3CA as a robust and promising biomarker for this condition. Moreover, we have also identified an immune-related regulatory axis involving XIST and OIP5-AS1/miR-216a/PIK3CA, which may play a key role in regulating ferroptosis during AMI progression.

**Supplementary Information:**

The online version contains supplementary material available at 10.1186/s12872-023-03622-z.

## Introduction

As early as 1998, AMI has consistently maintained its status as the most common and lethal cardiac event globally [1]. AMI occurs when a supply artery is blocked, leading to reduced blood flow and insufficient oxygen supply to the myocardial tissue downstream of the blockage. This insufficient blood flow and oxygen supply result in myocardial injury. Following an AMI attack, the ischemic heart tissue undergoes inflammation, fibrosis, and irreversible necrosis of myocardium [2]. So far, there are many vital treatments clinically available to rescue the ischemic heart tissue [3, 4], but only a few AMI victims can benefit from them on account of individual differences in efficacy and hemorrhage-related complications. Therefore, identifying potential biomarkers, seeking the molecular mechanisms, and finding innovative therapeutic targets for AMI have become urgent affairs.

Ferroptosis is induced by intracellular iron-mediated oxidative stress, characterized by accumulations of lipid peroxides [5, 6]. The molecular mechanisms involved in ferroptosis include maladjustment of two major redox systems (lipid peroxidation and thiols), abnormal iron metabolism, and some critical enzymes (like Glutathione Peoxidase-4, GPX4) [7]. Ferroptosis was first described in the central nervous system [8], but current studies suggest that ferroptosis has an important regulatory role in AMI [9]. For example, inhibiting the Hif1a/Ptgs2 pathway can have a protective role in coronary embolization-induced myocardial injury through mitigating the harmful effects of ferroptosis [10]. Under cysteine deprivation, neonatal rat ventricular myocytes (NRVM) rapidly respond to ferroptosis induced by GPX4 inhibition, aggravating myocardial damage during AMI [11]. Therefore, inhibiting ferroptosis in cardiomyocyte can exert myocardial protective effects, investigating the precise regulatory mechanisms associated with ferroptosis in the context of AMI is crucial.

The advancement of high-throughput technology has facilitated the identification and exploration of non-coding RNAs, including long non-coding RNAs (lncRNAs), microRNAs (miRNAs) and circular RNAs (circRNAs), as promising targets for the prevention, diagnosis, and therapeutic intervention of ischemia/reperfusion (I/R) injury [12]. LncRNAs, as ceRNA of miRNAs, have been found to regulate inflammation, lipid metabolism, angiogenesis, and other biological functions in AMI by affecting the downstream mRNA at the transcriptional level [13]. Hundreds of lncRNAs have been shown to regulate various pathological processes in AMI, making them vital biomarkers with better sensitivity and specificity [14]. For example, lncRNA TUG1 can regulate ROS accumulation in cardiomyocytes by targeting miR-132-3p/HDAC3 axis [15]. Therefore, an in-depth exploration of the function and mechanism of lncRNAs may provide a scientific basis for cutting-edge therapies for AMI.

The perturbation of immune system regulation plays a crucial role as a pathological mechanism in AMI [16, 17], The modulation of immune cell activity determined the severity of lesions and prognosis of AMI. It is reported that CD4^+^/CD8^+^ effector T cell, NK cell, and B cell can promote chemokine production during plaque rupture in AMI [18]. Dysfunctional mitochondria have been found to be key players in inflammatory response [19], and alterations in mitochondrial morphology and metabolism are important processes in ferroptosis. This suggests that dysfunctional mitochondria may exert a crucial effect in linking AMI ferroptosis and immune cell infiltration. However, to date, the ferroptosis-related biomarkers with immune infiltration in AMI have not been analyzed. Consequently, the assessment of ferroptosis-related biomarkers and their correlation with immune infiltration during the progression of AMI holds paramount importance in the context of advanced targeted therapeutics.

In our study, DE-FRGs were identified between AMI and normal groups from the GEO datasets. Firstly, we constructed two ceRNA networks related to ferroptosis of AMI according to datasets and targeted gene prediction analysis. By using integrated analysis among the ceRNA network, PPI network, and LASSO regression, key DE-FRGs was identified. The receiver operating characteristic (ROC) curve was used to evaluate diagnostic capabilities of these key DE-FRGs between AMI and control samples. We utilized the GSE59867 dataset for analyzing disparities in immune cell infiltration between samples from patients with AMI and normal samples. Spearman analysis confirmed the correlation between key DE-FRGs and immune cells. Eventually, the potential ceRNA regulatory axis related to the immune system in AMI was identified.

## Method

### Data collection and processing

**Fig. 1:**
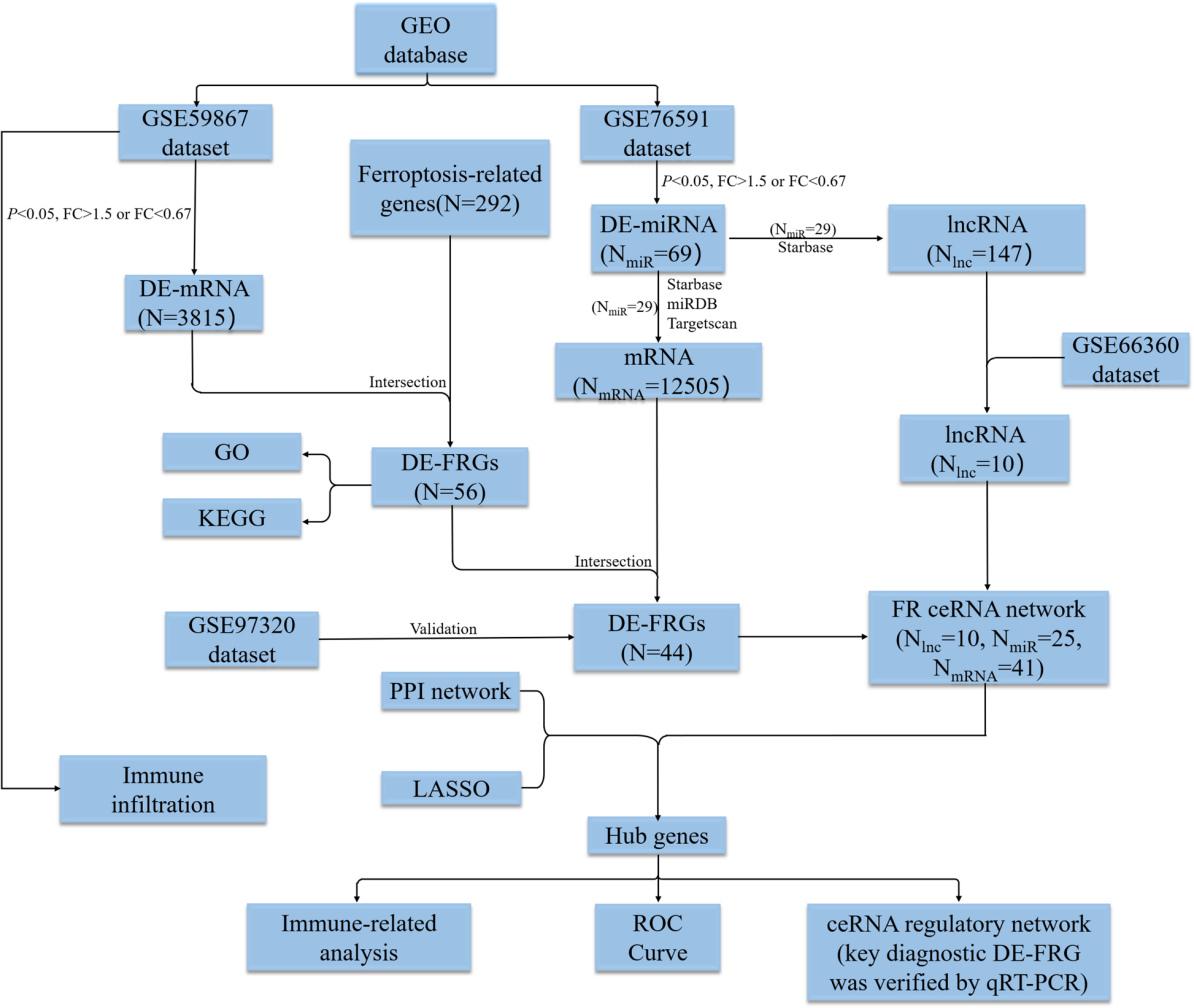
The workflow and data preprocessing of the overall study. DE-lncRNAs, differentially expressed lncRNAs; DE-miRNAs, differentially expressed miRNAs; DE-mRNAs, differentially expressed mRNAs; DE-FRGs, differentially expressed ferroptosis-related genes; PPI, protein–protein interaction; LASSO, least absolute shrinkage and selection operator

The miRNA, mRNA and lncRNA sequence datasets were obtained from the GEO database (http://www.ncbi.nlm.nih.gov/geo), which is a publicly accessible repository. To retrieve relevant datasets, we used the following search criteria: “acute myocardial infarction” AND “Homo sapiens”. Five microarray datasets were identified that consist of samples from both individuals with AMI and healthy controls, providing valuable information for our study. These datasets include GSE59867, GSE97320, GSE76591, GSE168149, and GSE66360, and they contained a total of 460 AMI samples and 120 healthy control samples. 292 ferroptosis-related genes (FRGs) were sourced from the FerrDb (http://www.zhounan.org/ferrdb) database and previous scientific publications [20–23]. This study utilized a range of bioinformatics techniques and statistical analyses to identify and characterize differentially expressed genes and relevant regulatory axis that are associated with ferroptosis in AMI. The detailed work procedure and data preprocessing steps for this study are outlined in Fig. 1.

### Differential expression gene analysis

As an online tool available at NCBI (https://www.ncbi.nlm.nih.gov/), GEO2R helps us to identify differentially expressed microRNAs (DE-miRNAs) and mRNAs (DE-mRNAs) between AMI samples and control samples. “Limma” packages was also used to assess sample distribution and data reliability of datasets. The volcano plot was utilized for visualizing the DE-miRNAs and DE-mRNAs in the GSE76591 and GSE59867 datasets. 292 ferroptosis-related genes were then cross-referenced with the DE-mRNAs to obtain DE-FRGs in AMI. Additionally, two heatmaps were separately generated to illustrate the inter-group expression of the DE-FRGs and DE-miRNAs, which provides a clear picture of the overall patterns of gene expression. The thresholds were set to *P* < 0.05 and fold change (FC) > 1.5 or FC < 0.67.

### Immune infiltration analysis

To clarify the role played by immune cells during AMI and their regulatory role, we utilised the ImmucellAI tool (https://bioinfo.life.hust.edu.cn/web/ImmuCellAI/) to perform immune cell infiltration analysis on dataset GSE59867. The ImmucellAI tool uses the ssGSEA method to calculate the enrichment fraction of individual samples in the gene expression profile and to estimate the relative proportion of the 24 immune cell subpopulations in each sample [24]. The results were presented in a bar plot and a correlation heatmap, which showed the differences in immune cell infiltration between AMI and control samples. The statistical significance of the results was determined using a cut-off value of *P* < 0.05.

### Functional enrichment analysis

To understand the biological properties and potential functions of the DE-FRGs, bioinformatics (http://www.bioinformatics.com.cn/), an online platform, was used for functional enrichment analysis [25]. Specifically, we performed Gene Ontology (GO) functional enrichment analysis (biological process (BP), molecular function (MF) and cellular component (CC)), as well as Kyoto Encyclopedia of Genes and Genomes (KEGG) pathway analysis [26, 27]. Bubble and histograms are used to present the results of GO/KEGG analysis.

### Construction of ceRNA network

To better understand the regulatory mechanisms associated with ferroptosis in AMI, we performed the construction of two ferroptosis-related ceRNA networks by means of database prediction and data overlap. Starbase database (v3.0, https://starbase.sysu.edu.cn/index.php) was used to predict lncRNAs that interacted with DE-miRNAs. GSE66360 dataset was used to verify predicted lncRNAs to obtain the intersection lncRNAs. Starbase, TargetScan (v7.2, http://www.targetscan.org/vert_72/) and miRDB (v6.0, http://mirdb.org/) was used to predict target mRNAs of DE-miRNAs, the predicted target mRNAs were overlapped with the previously identified DE-FRGs to obtain the overlapped DE-FRGs. GSE97320 dataset was used to verify the retained overlapped DE-FRGs. Ultimately, by leveraging the interplay between mRNA, miRNA, and lncRNA molecules, we employed Cytoscape software to construct ceRNA networks [28].

### Protein–protein interaction analysis

In order to clarify the interactions between the proteins translated by the DE-FRGs, a PPI network was generated using the STRING database (https://string-db.org/) [29]. A threshold of 0.4 (medium confidence) was set to determine significant interactions. Next, Minimal Common Oncology Data Elements (MCODE) plugin was used to identify significant clusters in the PPI network [30], The screening criteria for this cluster score were: degree cut-off = 3, node score cut-off = 0.2, k-core = 2, and max depth = 100. The cytoHubba plugin [31] was used to identify hub genes by Cytoscape. We employed five algorithms, specifically Maximal Clique Centrality (MCC), Density of MNC (DMNC), Maximum Neighborhood Component (MNC), Degree, and EcCentricity, to evaluate the top 10 hub genes [32]. All of the approaches have been approved by scholars and have been used in articles [33, 34].

### Screening of key DE-FRGs biomarker

Least absolute shrinkage and selection operator (LASSO) regression model uses a penalty function to reduce the coefficients of the regression model towards zero. This helps to select the most important variables and avoid overfitting [35]. LASSO was used to identify a subset of genes that are most closely associated with AMI outcome prediction in this study. The LASSO-selected genes were overlapped with the hub genes in PPI network and DE-FRGs from ceRNA networks to identify key DE-FRGs biomarkers for distinguishing AMI patients from controls.

### Immune-related analysis

To further investigate the potential association between the key DE-FRGs biomarker and immune cell subpopulations in AMI, we performed Spearman correlation analysis using an expression data matrix of DE-FRGs. The correlation coefficient and* P*-value were calculated for the key DE-FRGs and 24 immune cell subpopulations. The results were then visualized using a lollipop chart, which correlation coefficients shown as the length of the sticks and *P*-values shown as the colour intensity. A *P*-value < 0.05 was considered statistically significant.

### Diagnostic performance of key DE-FRGs biomarker in AMI

The ROC curve illustrates the diagnostic performance test by depicting the trade-off between sensitivity (true positive rate) and specificity (true negative rate). The AUC is the area under the ROC curve and the closer the value of the AUC is to 1, the more reliable the factor is for the diagnosis of the disease. In our study, we used the area under the ROC curve to evaluate the discriminative power of the key DE-FRGs biomarker in distinguishing AMI patients from healthy controls. The 95% CI provides a range of values within which the true AUC is likely to lie [36].

### H9C2 cardiomyocyte culture and reverse transcription polymerase chain reaction analysis (qRT-PCR)

H9C2 cells was purchased from the American Model Species Collection Center (ATCC) and was cultured in a constant temperature incubator at 37℃ and 5% CO_2_. An in vitro hypoxic cardiomyocyte model was constructed using a hypoxic incubator (Billups Rothenberg) with 5% CO_2_ and 95% nitrogen. The H9C2 cells were inoculated into a six-well plate and were subjected to hypoxia treatment. When the cells grow to about 80–90%, the total RNA was isolated and extracted by TRIzol reagent (Invitrogen). A 20ul reverse transcription reaction system mixture was prepared for cDNA synthesis at 50 °C, 30 min, 75 °C, and 5 min. The real-time PCR reaction was then performed on the real-time PCR instrument 7500 (ABI). The control gene of glyceraldehyde-3-phosphate dehydrogenase (GAPDH) was used as the control gene, and the relative expression of the target gene was calculated by the method of 2^−ΔΔCt^ [37]. The PCR primer sequences were designed by Invitrogen. The primer sequences for PIK3CA are as follows: forward: 5’-AGGATGCCCAACTTGATGCTGATG-3’ and reverse: 5’-CCGTTCATATAGGGTGTCGCTGTG-3’. The primer sequences for GAPDH are as follows: forward: 5’-CTGGAGAAACCTGCCAAGTATG-3’ and reverse: 5’-GGTGGAAGAATGGGAGTTGCT-3’.

## Statistical analysis

Gene expression variability analysis of microarray data was performed using the GEO2R (GEO2R, http://www.ncbi.nlm.nih.gov/geo/geo2r/) platform, which is based on the R language and meets statistical criteria. *P* < 0.05 and FC > 1.50 or FC < 0.67 were considered as screening criteria for significantly different genes, which is consistent with previous papers' analysis methods [38, 39].

## Results

### Identification of 56 DE-FRGs

**Fig. 2:**
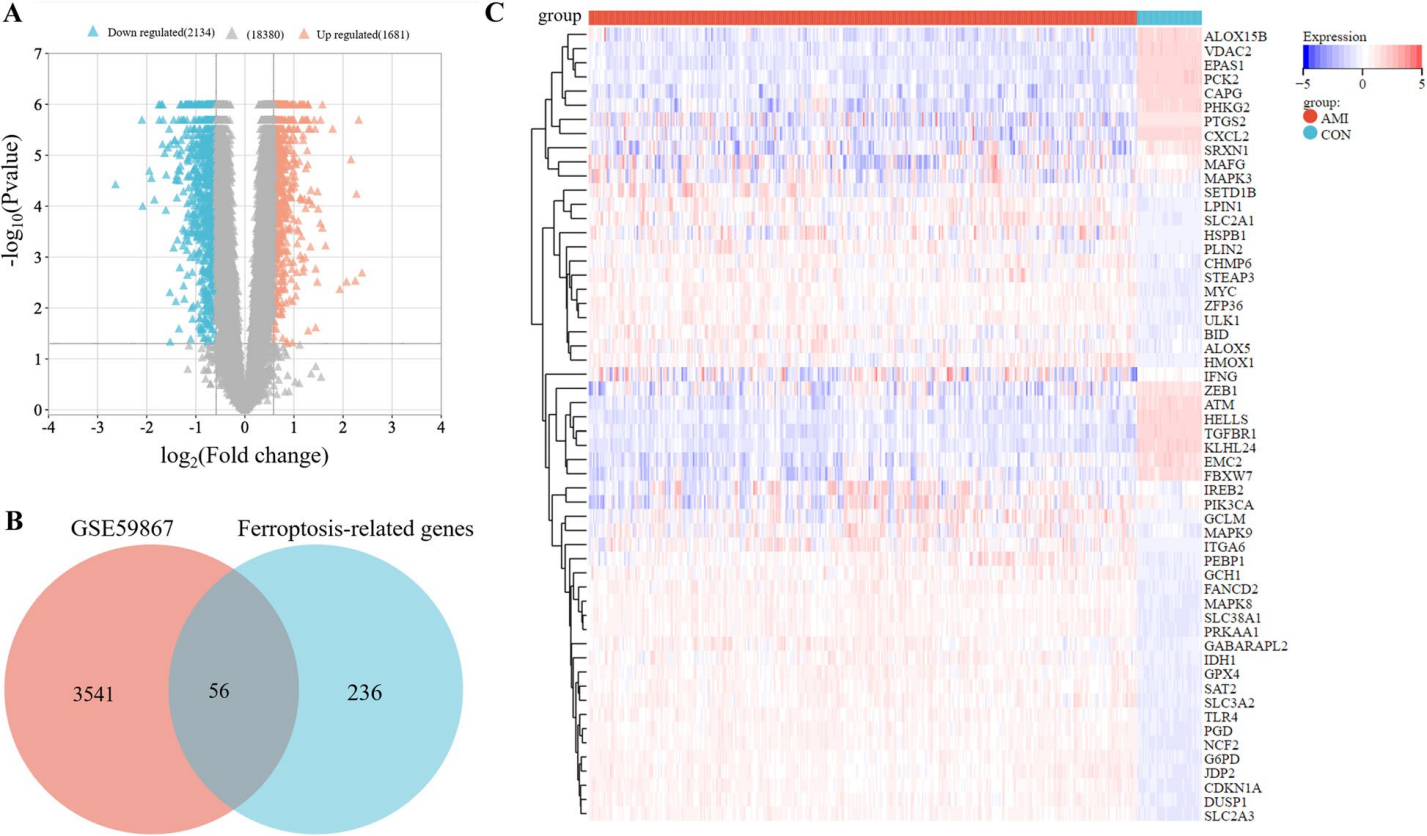
The gene expression data of DE-mRNAs between AMI samples and control samples. **A** Volcano plot corresponding to the expression profile of DE-mRNAs in GSE59867 dataset. The pink dots represent up-regulated genes, the grey dots represent non-significant genes, the blue dots represent down-regulated genes. **B** 2 set Venn diagram shows the integration strategy among GSE59867 dataset and ferroptosis-related genes. The blue circle represents for ferroptosis-related genes, the red circle represents for DE-mRNAs in GSE59867 dataset. As shown, there were 56 DE-FRGs. **C** Cluster heatmap for 56 DE-FRGs in GSE59867 dataset

This study included a total of 580 patients, consisting of 120 control samples and 460 AMI samples. The elaborated information of the 5 datasets used in the study is presented in Table 1. The volcano plot in Fig. 2A shows the data distribution of the GSE59867 datasets, a total of 3821 differentially expressed mRNAs (*P* < 0.05) were identified (1,681 up-regulated and 2,134 down-regulated). By analyzing GSE59867 and 292 ferroptosis-related genes, we identified 56 DE-FRGs (Fig. 2B), and exhibited the expression of DE-FRGs using a heatmap (Fig. 2C). The thresholds for significant differences were set at *P* < 0.05 and FC > 1.50 or FC < 0.67. The results of sample normalization and distribution of the dataset are shown in Supplementary Material Figure 1-5.

**Table 1:** Comprehensive details regarding the gene expression profiles investigated in this study are provided below

Dataset	Platform	Experiment type	Control	AMI	Contury	Submission	Samples	Application
GSE59867	GPL6244	Expression profiling by array	46	390	Poland	2015	Peripheral blood	Identification for DE-mRNAs
GSE76591	GPL16384	Non-coding RNA profiling by array	12	9	Japan	2019	Human heart tissue	Identification for DE-miRNAs
GSE97320	GPL570	Expression profiling by array	3	3	China	2019	Peripheral blood	Validation for 44 DE-FRGs
GSE66360	GPL570	Expression profiling by array	50	49	La Jolla	2019	Circulating endothelial cells	Validation for DE-lncRNAs
GSE168149	GPL19117	Non-coding RNA profiling by array	9	9	Germany	2021	Monocyte	Validation for DE-miRNAs

### Immune infiltration landscapes

**Fig. 3:**
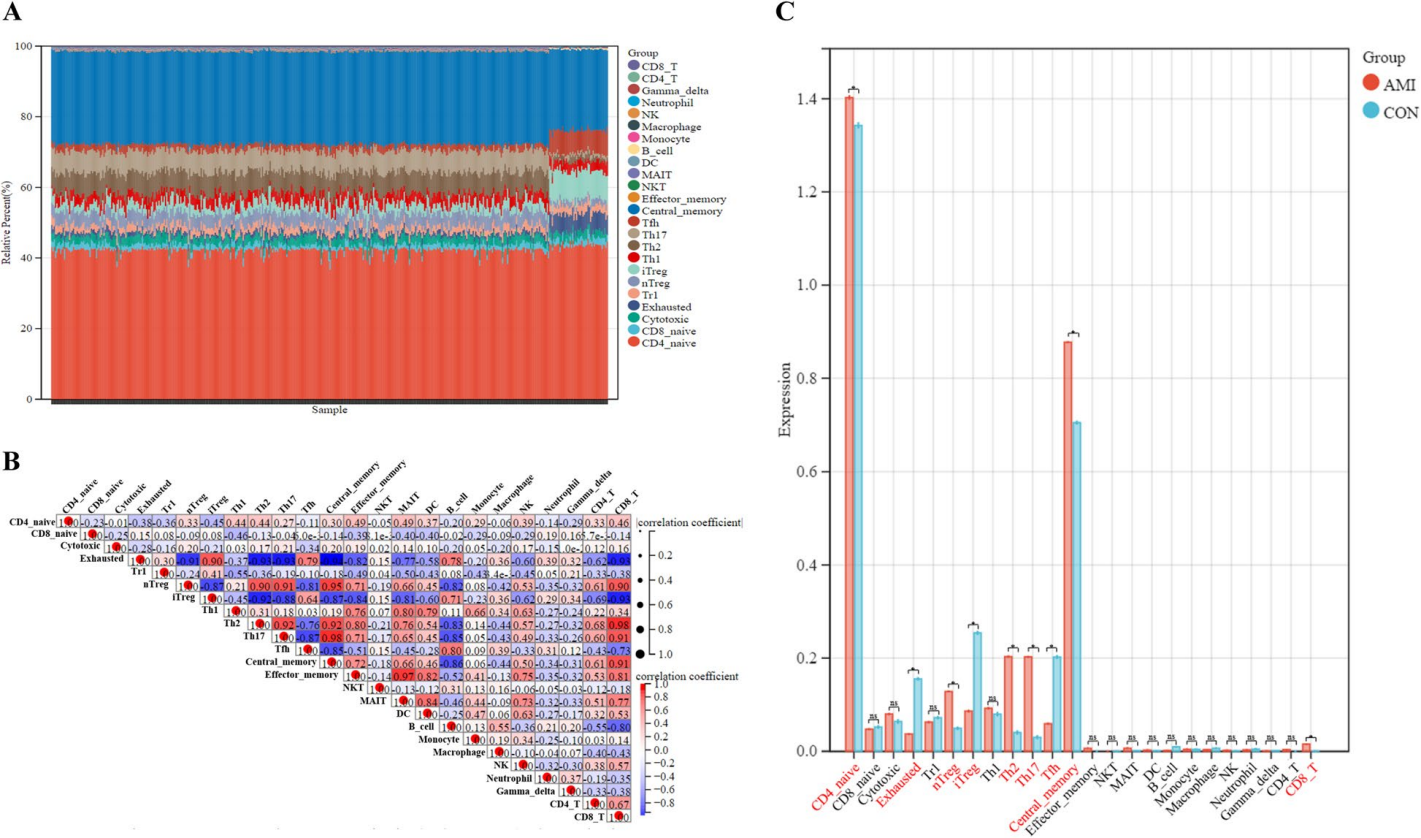
Results of immune infiltration analysis. **A** The stack bar diagram displays the relative percent of 24 immune cell sub-populations in each sample. **B** Correlation heatmap of 24 immune cell sub-populations. The red represents positive correlation and the blue represents negative correlation. **C** Bar diagram displays different fractions of 24 immune cell sub-populations in AMI and control samples

To gain a better understanding of the roles of immune cells in the AMI cardiac microenvironment, we investigated the immune cell landscapes between AMI tissues and controls in the dataset GSE59867. For the results of immune infiltration, we used stacked bar plots to clearly show the proportion of the 24 immune cell subsets in each sample (Fig. 3A). The correlation heatmap between the 24 immune cell subpopulations in AMI showed that central-memory T cells were negatively correlated with exhausted T cells, while being positively correlated with Th17 cells. NK T cells and CD8 T cells respectively displayed positive correlations with MAIT cells and Th2 cells (Fig. 3B). The bar diagram showed that compared with control samples, CD4_native cells, nTreg cells, Th2 cells, Th17 cells, central-memory cells, and CD8_T cells were all presented with higher infiltrates in AMI samples, but exhausted cells, iTreg cells, and Tfh cells were all presented with lower infiltrates in AMI samples (Fig. 3C).

### Functional enrichment analysis of 56 DE-FRGs

**Fig. 4:**
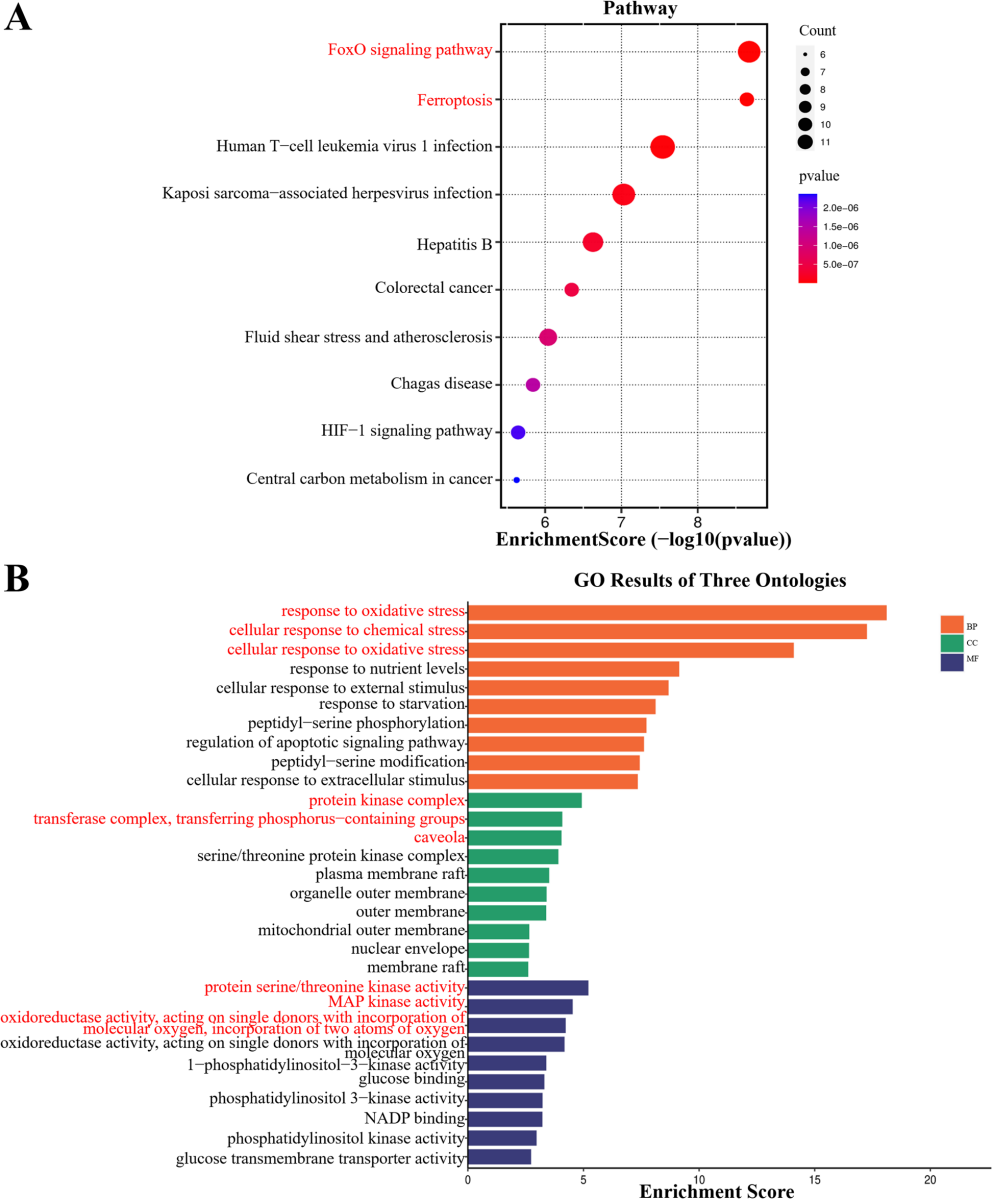
Functional enrichment results of 56 DE-FRGs. **A** Significant enriched KEGG pathways analysis for 56 DE-FRGs. **B** Significant enriched GO terms for 56 DE-FRGs. (CC, cellular component; BP, biological process; MF, molecular function)

To clarify the regulatory role and the signaling pathways that the 56 DE-FRGs functions in the organism, we performed GO/KEGG analysis on the 56 DE-FRGs. The KEGG pathway analysis revealed that these genes are mainly involved in the FoxO signaling pathway and ferroptosis (Fig. 4A). The analysis revealed that the 56 DE-FRGs were predominantly enriched in biological processes (BP) related to the response to oxidative stress, cellular response to chemical stress, and cellular response to oxidative stress. Regarding cellular components (CC), the 56 DE-FRGs were associated with protein kinase complex, transferase complex, transferring phosphorus-containing groups and caveola. Regarding molecular function (MF), the 56 DE-FRGs were mainly linked to protein serine/threonine kinase activity, MAP kinase activity, and oxidoreductase activity, acting on single donors with incorporation of molecular oxygen, incorporation of two atoms of oxygen (Fig. 4B).

### Construction of ceRNA network

**Fig. 5:**
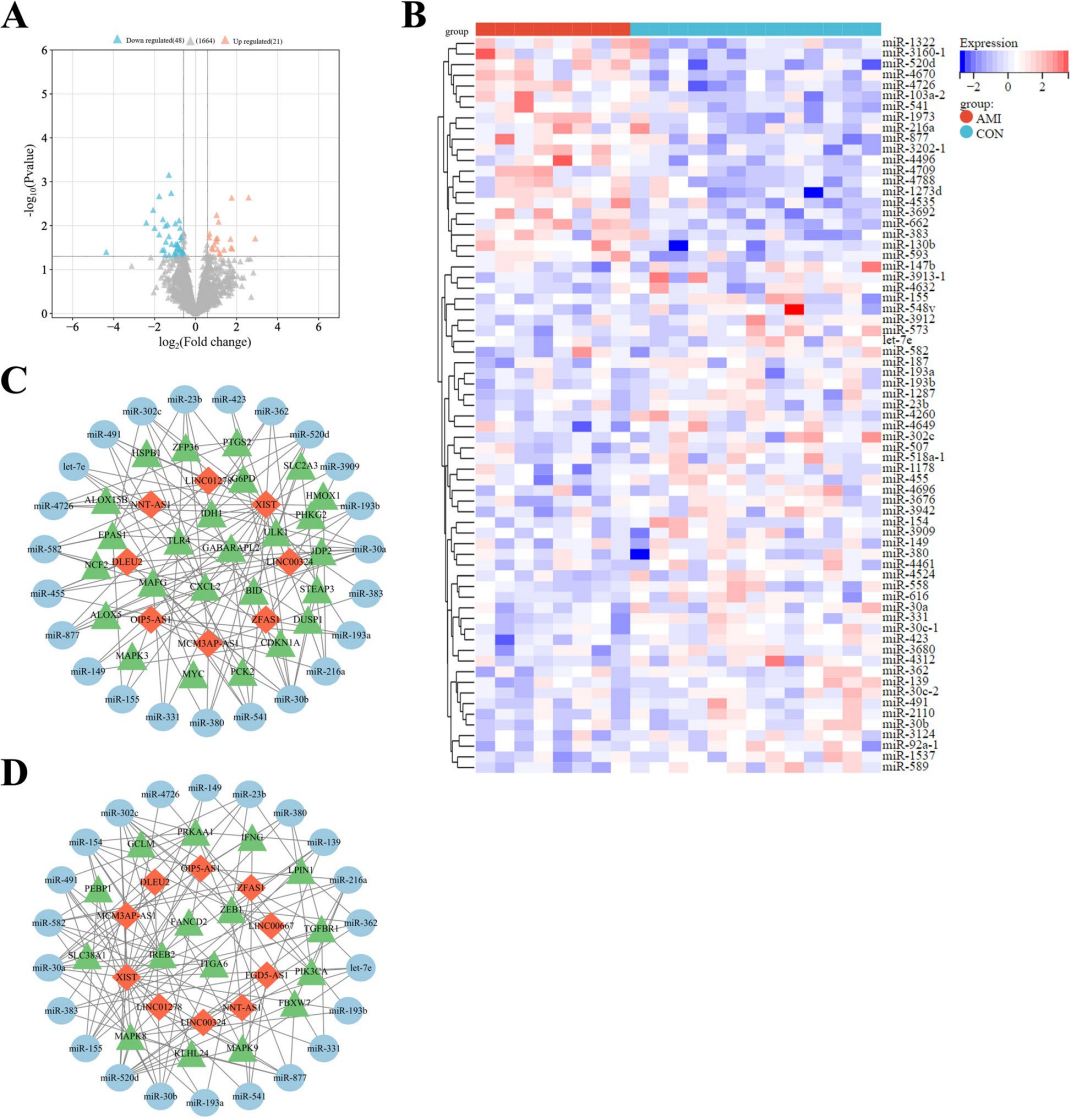
The gene expression data of DE-miRNAs between AMI samples and control samples. **A** Volcano plot corresponding to the expression profile of DE-miRNAs in GSE76591 dataset. The pink dots represent up-regulated genes, the grey dots represent nonsignificant genes, the blue dots represent down-regulated genes. **B** Cluster heatmap for DE-miRNAs in GSE76591 dataset. **C** The first ceRNA network is constructed via 25 up-regulated DE-FRGs (green triangle) in AMI, their 23 corresponding miRNAs (blue ellipse) as well as 8 lncRNAs (red rhombus), which was composed of 56 nodes and 102 edges. **D** The second ceRNA network is constructed via 16 down-regulated DE-FRGs (green triangle) in AMI, their 22 corresponding miRNAs (blue ellipse) as well as 10 lncRNAs (red rhombus), which was composed of 48 nodes and 121 edges

The volcano plot in Fig. 5A shows the data distribution of the GSE76591 dataset, with a total of 1731 miRNAs identified (*P* < 0.05). Then, 69 DE-miRNAs were directly identified in the GSE76591 dataset (21 up-regulated miRNAs and 48 down-regulated miRNAs), the screening criteria was set to *P* < 0.05 and FC > 1.50 or FC < 0.67. The heatmap demonstrates the inter-group differences of 69 DE-miRNAs in their expression levels (Fig. 5B). The Starbase database predicted that 29 DE-miRNAs had binding sites with 147 lncRNAs among the identified 69 DE-miRNAs. Furthermore, combining the predictions from miRDB, TargetScan, and Starbase databases revealed that 64 DE-miRNAs had binding sites with downstream mRNAs among the identified 69 DE-miRNAs. Further analysis revealed that only 29 DE-miRNAs had binding sites with both 147 lncRNAs and 12,505 mRNAs. We then merged these predictive mRNAs with 56 DE-FRGs, identifying 44 overlapped DE-FRGs. We validated these 44 overlapped DE-FRGs using the GSE97320 dataset and 10 lncRNAs using the GSE66360 dataset. Based on the validation results, 44 DE-FRGs, 29 miRNAs and 10 lncRNAs were retained to construct ceRNA network by Cytoscape. The first ceRNA network was constructed using 25 up-regulated DE-FRGs, 8 lncRNAs and 23 corresponding miRNAs, with 56 nodes and 102 edges (Fig. 5C). The second ceRNA network was constructed using 16 down-regulated DE-FRGs, 10 lncRNAs and 22 corresponding miRNAs, with 48 nodes and 121 edges (Fig. 5D). The two ceRNA networks included a total of 10 lncRNAs, 25 miRNAs, and 41 DE-FRGs (Table 2).

**Table 2:**
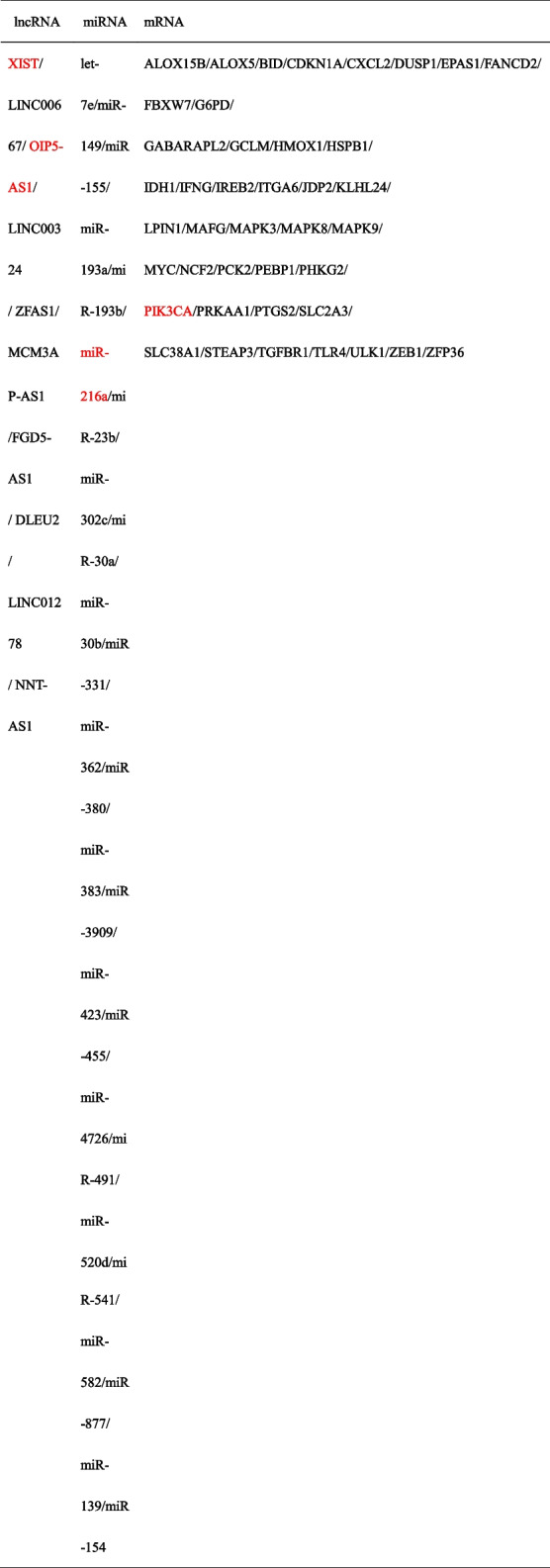
The lncRNAs, miRNAs AND mRNAs in ceRNA network

The red font represents the lncRNA, miRNA and mRNA contained in final immune-related ceRNA axis in AMI. And PIK3CA was identified as a significant ferroptosis-related biomarker in AMI

### PPI network analysis and CytoHubba gene identification

**Fig. 6:**
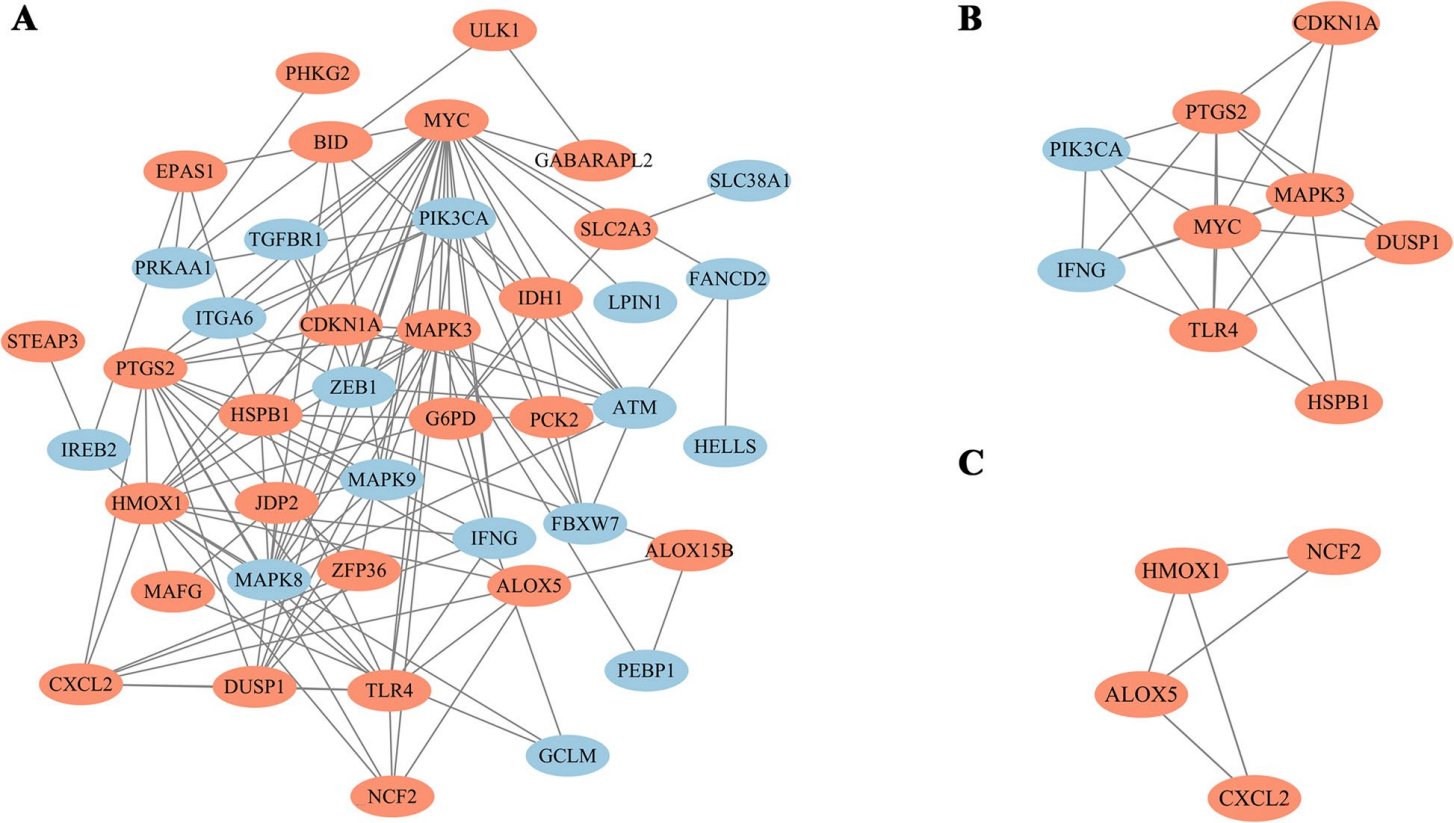
PPI network (**A**) The interaction network between proteins coded by DE-FRGs was composed of 42 nodes and 124 edges. Each node represents a protein, whereas each edge represents one protein–protein association. **B**-**C** Cluster plots represent the interaction network identified by MCODE. The red filled ellipses represent down-regulated genes, and blue filled ellipses represent up-regulated genes

The PPI network of 44 shared DE-FRGs showed that there were 42 nodes (representing proteins) and 124 edges (representing interactions between the proteins) (Fig. 6A). We then further analyzed the PPI network using the MCODE plugin and found that there were 2 clusters in the network, containing a total of 13 DE-FRGs (Fig. 6B and C). The DE-FRGs in these two clusters possessed a much closer interaction relationship. We identified the top 10 genes among 42 DE-FRGs by using the cytoHubba plugin, and MAPK3 (mitogen-activated protein kinase 3), TLR4 (toll-like receptor 4) and PIK3CA (phosphatidylinositol-4,5-bisphosphate 3-kinase catalytic subunit alpha) were cross-checked by five algorithms (Table 3). This suggest that MAPK3, TLR4 and PIK3CA potentially have significant involvement in the pathogenesis of AMI.

**Table 3:**
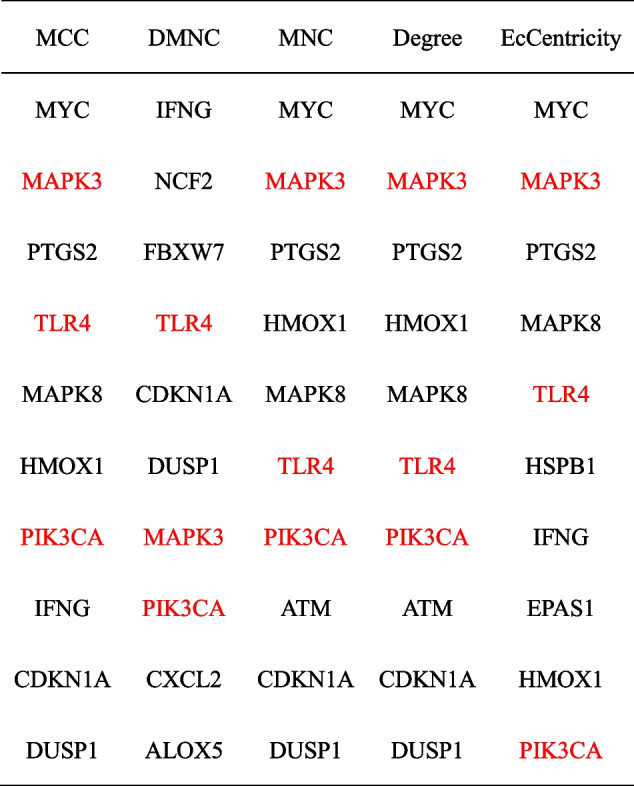
The hub genes identified by using five different algorithms of cytoHubba

Three hub genes were identified by cross-checking the results of five algorithms. And three hub genes were marked in red font

### Screening for key DE-FRG biomarkers

**Fig. 7:**
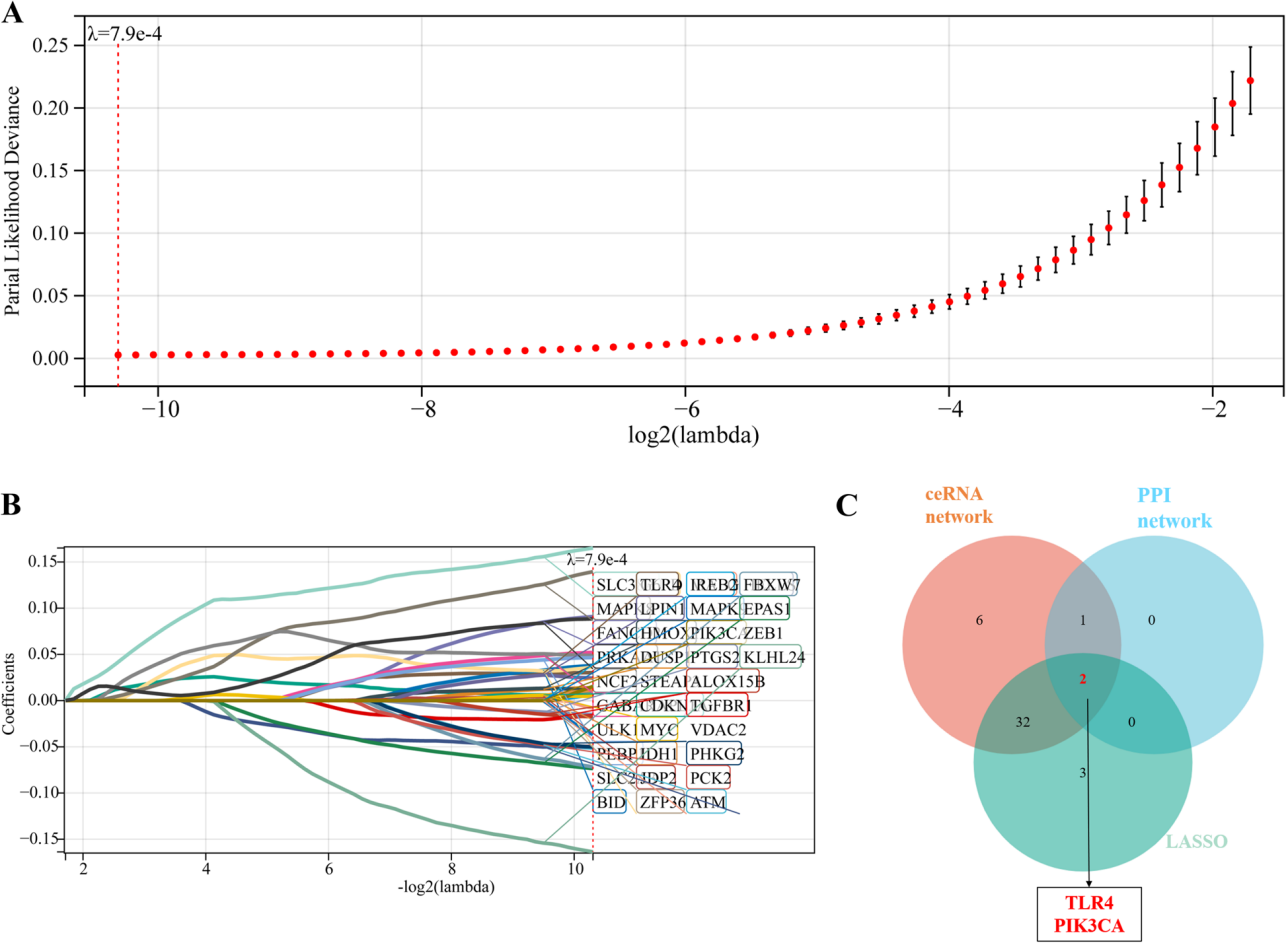
LASSO regression (**A**) Ten time cross-validation for tuning parameter selection in the LASSO model. **B** LASSO coefficient profiles. The method uses an λ penalty to shrink some regression coefficients to exactly zero. The binomial deviance curve was plotted versus -log (λ), where λ is the tuning parameter. **C** 3 set Venn diagram shows the integration strategy among ceRNA, PPI and LASSO regression. The red circle represents for ceRNA, the blue circle represents for PPI, the green circle represents for LASSO regression. As shown, there were 2 key DE-FRGs (TLR4, PIK3CA)

To identify the most promising diagnostic gene biomarkers, the LASSO regression algorithm was applied to the 44 retained DE-FRGs. When “lambda.min = 0.00079”, partial likelihood deviance is minimal, the model fits well, 37 genes were obtained, including ATM, CDKN1A, HELLS, ZEB1, MAFG, PTGS2, SLC38A1, TGFBR1, TLR4, ZFP36, ALOX15B, BID, DUSP1, EPAS1, PEBP1, ULK1, FANCD2, FBXW7, G6PD, GABARAPL2, HMOX1, IDH1, IREB2, JDP, LPIN1, MAPK8, MAPK9, MYC, NCF2, PCK2, PHKG2, PIK3CA, PRKAA1, SLC2A3, STEAP3 and VDAC2 (Fig. 7A and B). After the 37 genes were then intersected with the 41 shared DE-FRGs in ceRNA and the 3 hub genes in PPI, we obtained 2 key DE-FRGs: TLR4 and PIK3CA (Fig. 7C). Table 4 provided more detailed information about TLR4 and PIK3CA.

**Table 4:** More information about the 2 key DE-FRGs

Gene	Full name	Protein coded	Role	logFC	*P* value
TLR4	toll like receptor 4	toll like receptor 4	Driver	0.3383	< 0.05
PIK3CA	phosphatidylinositol 4,5-bisphosphate 3-kinase catalytic subunit alpha	phosphatidylino-sitol 3-kinases	Driver	-0.2377	< 0.05

### Immune-related analysis

**Fig. 8:**
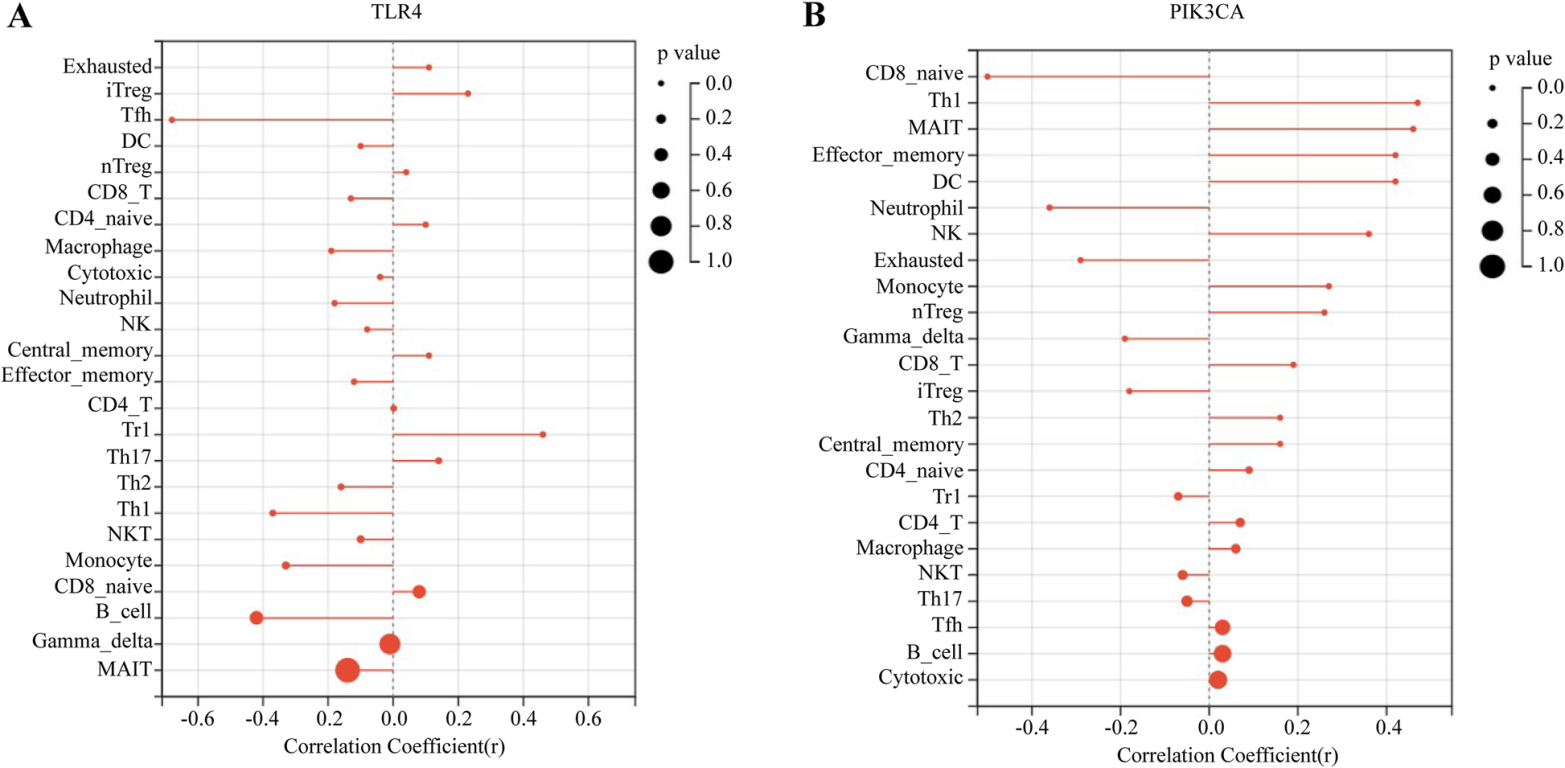
Correlation between TLR4/PIK3CA and 24 immune cells. **A** Correlation analysis between immune cell subpopulations and TLR4. **B** Correlation analysis between immune subpopulations and PIK3CA. The dot with a smaller size has a smaller *p* value. The X axis represents correlation coefficient

Spearman correlation analysis revealed a substantial relationship between PIK3CA/TLR4 and several subpopulations of infiltrating cells. Specifically, TLR4 had a negative correlation with Tfh cells (*r* = -0.68, *P* < 0.05), B cells (*r* = -0.42, *P* < 0.05), and Th1 cells (*r* = -0.37, *P* < 0.05), while having a positive correlation with Tr1 cells (*r* = 0.46, *P* < 0.05) (Fig. 8A). PIK3CA had a negative correlation with CD8-naive cells (*r* = -0.5, *P* < 0.05), while having a positive correlation with Th1 cells (*r* = 0.47, *P* < 0.05), Effector-memory T cells (*r* = 0.42, *P* < 0.05), and MAIT cells (*r* = 0.46, *P* < 0.05) (Fig. 8B). These results suggested that PIK3CA/TLR4 could partly reflect the condition of the cardiac microenvironment in AMI.

### Diagnostic performance of key DE-FRGs biomarkers in AMI

**Fig. 9:**
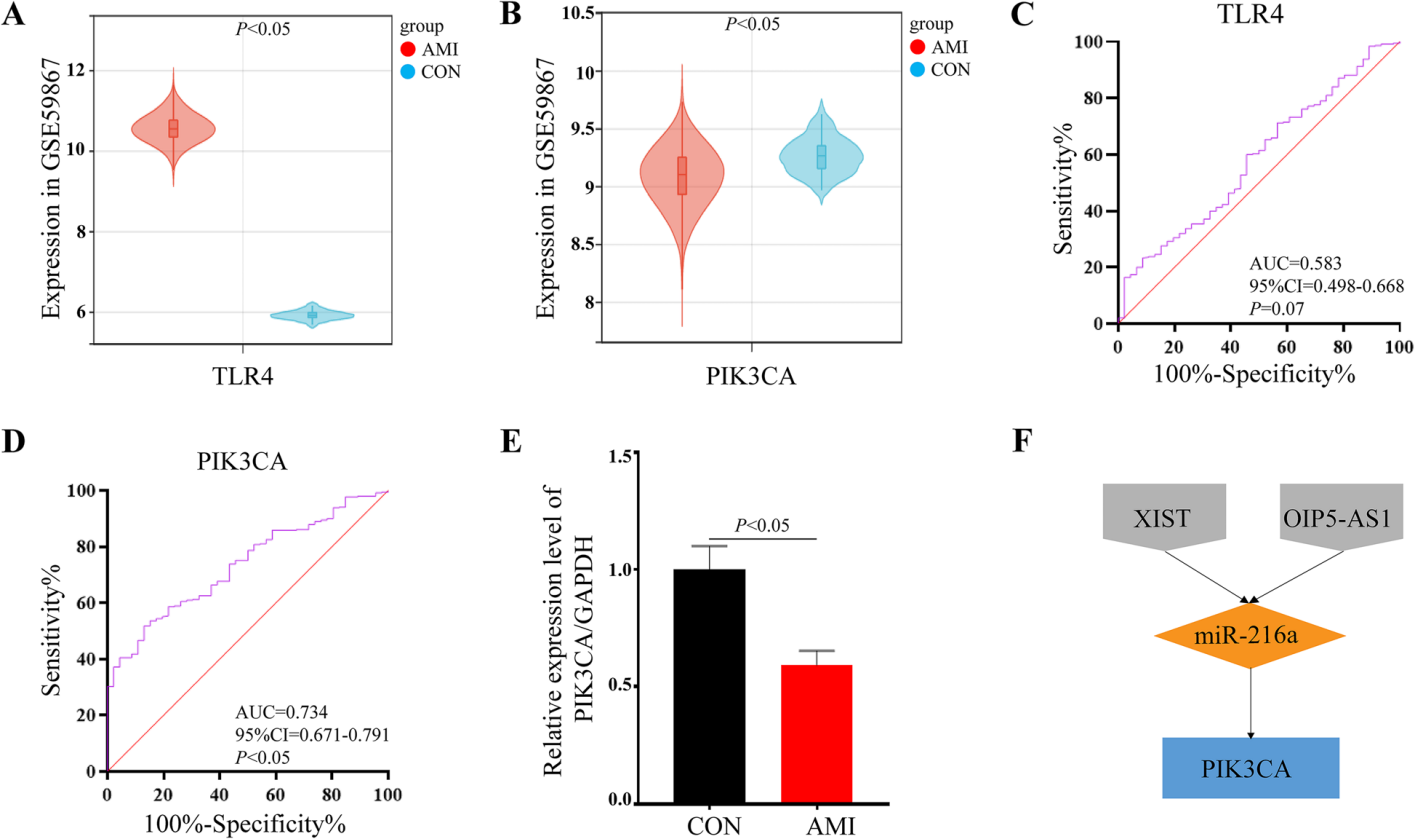
Diagnostic performance of TLR4 and PIK3CA. **A** The violin plot represents the expression of TLR4 in the dataset GSE59867. The red mark represents AMI samples, the blue mark represents control samples. **B** The violin plot represents the expression of PIK3CA in the dataset GSE59867. The red mark represents AMI samples, the blue mark represents control samples. **C** Receiver operating characteristic (ROC) curve for TLR4. **D** ROC for PIK3CA. **E** The qRT-PCR results of PIK3CA in H9C2 rat cardiomyocytes. **F** XIST and OIP5-AS1/miR-216a/PIK3CA ceRNA network

TLR4 and PIK3CA have been identified as key DE-FRGs biomarkers with potential diagnostic value for AMI. In comparison to control group, the expression of TLR4 was found to be significantly up-regulated in the AMI group in GSE59867 dataset (*P* < 0.05) (Fig. 9A), Conversely, the expression of PIK3CA was observed to be significantly decreased in the AMI group in GSE59867 dataset (*P* < 0.05) (Fig. 9B). To evaluate the diagnostic performance of TLR4 and PIK3CA, ROC analysis was performed using TLR4 and PIK3CA expression data from the GSE59867 dataset. The AUC of TLR4 was 0.583 (95%CI = 0.498–0.668, *P* = 0.07) (Fig. 9C), which suggests that it may not be a strong diagnostic biomarker for AMI. However, the AUC of PIK3CA was 0.734 (95%CI = 0.671–0.791, *P* < 0.05) (Fig. 9D), indicating that it has good diagnostic potential for AMI. Meanwhile, we constructed a rat cardiomyocyte hypoxia model in vitro and used qRT-PCR to detect the relative expression level of PIK3CA. The result of qRT-PCR was consistent with the GSE59867 database: the expression level of PIK3CA in H9C2 hypoxic cardiomyocytes decreased significantly compared with the control group (*P* < 0.05, FC = 0.59) (Fig. 9E). These findings indicate that PIK3CA holds promise as a potential diagnostic biomarker for AMI.

### LncRNA-XIST and OIP5-AS1/miR-216a/PIK3CA axis

**Fig. 10:**
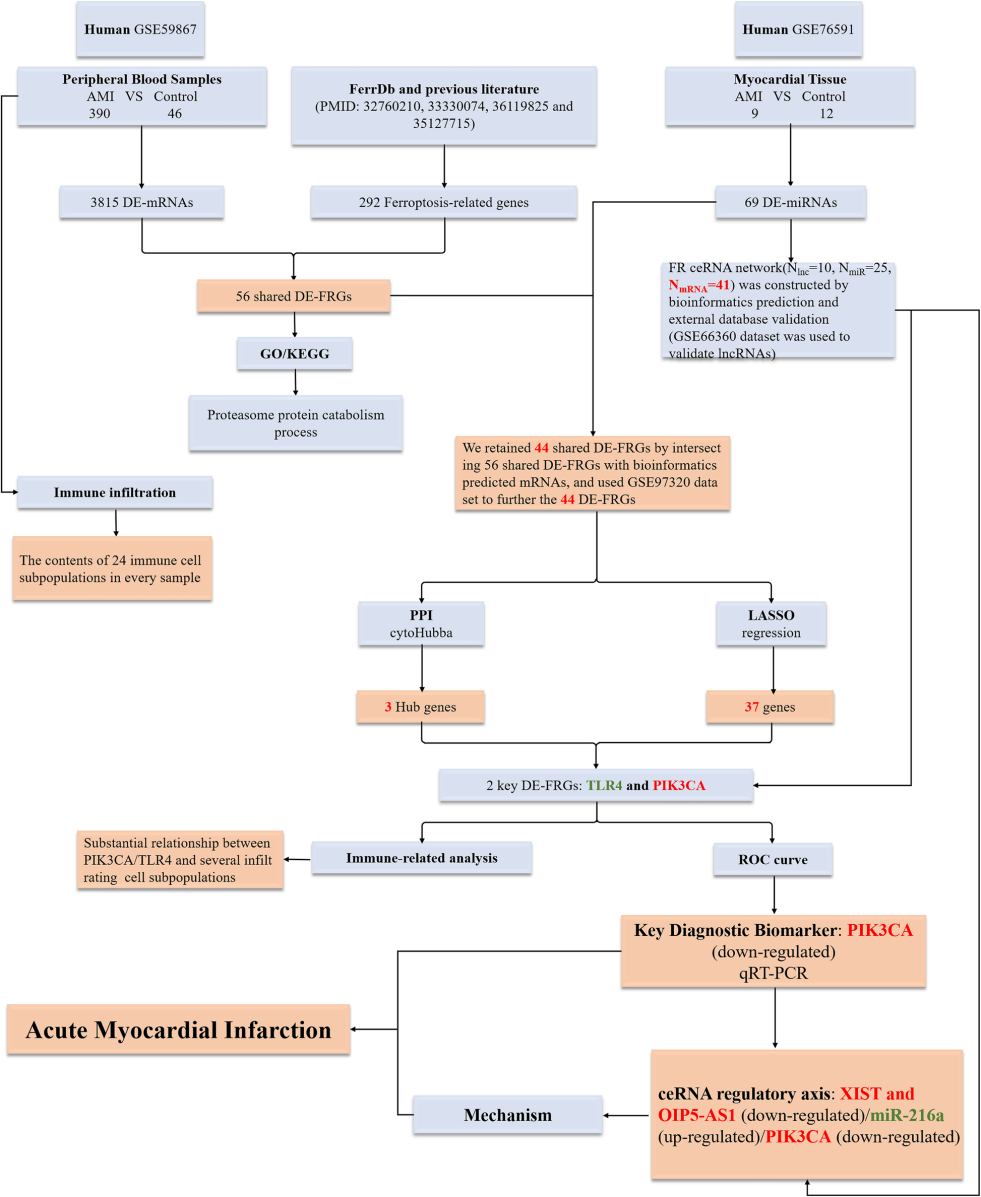
The technical workflow of this article. The red word means down-regulation, and the green word means up-regulation

To identify the regulatory axis of PIK3CA biomarker in AMI, we employed a ceRNA network analysis approach. Since a single mRNA can interact with multiple miRNAs, we narrowed down the potential miRNAs by utilizing the GSE168149 dataset to ensure accuracy in our analysis. After validation, miR-216a was identified as a potential upstream miRNA that could regulate the expression of the PIK3CA. We also found that XIST and OIP5-AS1 were the top lncRNAs co-regulating miR-216a in the context of AMI, suggesting the existence of an intricate regulatory network in the pathogenesis of AMI (Fig. 9F). These novel findings shed light on the potential roles of PIK3CA biomarker in AMI and may provide new avenues for further research in this field. The schematic representation of our study’s workflow was depicted in Fig. 10.

## Discussion

Despite significant progress in AMI treatment in recent years, existing therapies such as thrombolytic therapy, medication and interventional therapy still have limitations. To address this, the potential roles of non-coding RNA in AMI have been extensively studied, particularly in relation to ferroptosis. Nevertheless, there remains a dearth of research regarding the specific regulatory mechanisms and immune infiltration landscapes associated with ferroptosis-related genes in AMI. Our study utilized an integration approach that combined ceRNA, LASSO, external dataset validation, and PPI network analysis to identify TLR4/PIK3CA as key DE-FRGs in AMI. And the ROC result showed PIK3CA was a robust and significant diagnostic biomarker in AMI. Furthermore, we analyzed immune infiltration using immucellAI.

In Table 5, we have presented two earlier studies that utilized bioinformatic analysis to identify crucial genes associated with AMI [40, 41]. Here, our study stands out by providing significant novel findings compared to two published papers. Firstly, we are the first to identify and explore the immune-related FRGs in the context of AMI. This represents a unique and previously unexplored aspect of AMI pathogenesis. Additionally, we have constructed and validated a novel ferroptosis-related ceRNA network involving XIST, OIP5-AS1, miR-216a, and PIK3CA. This ceRNA network differs from the previous studies, indicating a fresh perspective on the regulatory mechanisms underlying AMI.

**Table 5:** Several findings in this study exhibit substantial innovation in comparison to the published literature

Items	Identification of Hub Genes in AMI Based on Bioinfomatics Analysis		
	Our findings	PMID: 37115066	PMID: 35585822
Years	2023	2023	2023
Test set	GSE59867 and GSE76591	GSE95368	GSE76387 and GSE161427
Species/tissue	Human/heart tissue and peripheral blood	Human/peripheral blood	Mice/heart tissue
Key genes	Key DE-FRG diagnostic biomarker: PIK3CA	S100A9, MAPK3, MAPK1, MMP3, IL17A and HSP90AB1	Col5a1
Validation set	GSE97320, GSE66360 and GSE168149	-	-
Verification	qRT-PCR	qRT-PCR	Western blotting
Mechanism	LncRNA–miRNA–DE-FRG ceRNA network: XIST and OIP5-AS1/miR-216a/PIK3CA	-	-

Recent research has highlighted the role of Toll-like receptor 4 (TLR4) in regulating ferroptosis in myocardial tissue. Inhibition of TLR4 has been shown to alleviate heat stroke-induced cardiomyocyte injury through inhibiting ferroptosis [42], while TLR4 knock-down has been found to retard ferroptosis in rats with heart failure [43]. Although the precise mechanism through which TLR4 regulates ferroptosis in AMI is not yet fully understood, it has been shown to be associated with ROS generation in AMI [44]. Therefore, it is possible that TLR4 can regulate ferroptosis in AMI. However, the ROC results for TLR4 were not significant.

In addition, the gene PIK3CA, which encodes phosphatidylino-sitol 3-kinases (PI3ks) [45], has been implicated in the modulation of ferroptosis in various diseases, including melanoma [46], rheumatoid arthritis [47], and lung injury [48], primarily via the PI3K/AKT/mTOR signaling pathway. The regulatory mechanism between PI3Ks and ferroptosis in AMI is not yet clear, but studies have shown that PI3Ks can affect myocardial apoptosis and autophagy by regulating Akt in hypoxic reoxygenated myocardial injury [49]. Activation of PI3Ks has also been found to alleviate mitochondrial apoptosis in AMI rats [50]. Therefore, PIK3CA could be the critical gene to regulate ferroptosis in AMI, which could be a hot topic for future research.

To confirm that PIK3CA are key DE-FRGs, we employed GSE168149 dataset to validate its target miRNA, which we found to be miR-216a. miRNAs typically regulate their target genes by inhibiting the expression of mRNA or promoting its degradation [51], indicating a negative correlation between the two. Our results indicated that PIK3CA was down-regulated, indicating that miR-216a should be up-regulated. Although miR-216a was found to be up-regulated in GSE76591 dataset, there was limited literature on its role in AMI. To gain further insights, we also explored lncRNAs that bind to miR-216a and identified two consensus lncRNAs: XIST and OIP5-AS1 (XIST, FC = 0.0001, *P* < 0.05; and OIP5-AS1, FC = 0.1923, *P* < 0.05). Wu et al. confirmed that miR-216a and OIP5-AS1 have direct binding sites through a dual-luciferase reporter assay [52]. One study suggests that XIST may promote myocardial fibrosis after AMI by sponging miR-155-5p [53], but there is limited literature on the roles of miR-216a and XIST in AMI. To validate our findings and gain a deeper understanding of the potential roles of miR-216a, XIST, and OIP5-AS1 in the pathogenesis of acute myocardial infarction (AMI), additional research is required in the near future.

Ferroptosis has also been found to have a link with the immune system, as ferroptotic cells can identify and affect innate immune cells and adaptive immune cells, triggering a series of immune response [54]. Recognized as a critical signal on the surface of ferroptotic cells, 1-stearoyl-2–15-HpETE-sn-glycero-3-phosphatidylethanolamine (SAPE-OOH) has the capability to be acknowledged by the TLR2 receptor in macrophage [55]. And immune cells can also regulate ferroptosis, for example, LNC2 secreted by neutrophils can induce ferroptosis and accelerate tissue loss in lung cancer [56]. Claire’s study showed that M1 macrophages can exacerbate the expansion of infarct size, while M2 macrophages are helpful for myocardial repair and inflammation to subside [57]. Nevertheless, our analysis of immune cell infiltration using the GSE59867 dataset revealed that AMI samples exhibited lower levels of macrophage infiltration compared to the control samples. This implies that the macrophages more significantly affected by AMI are likely to be of the M2 type and that promoting infiltration of this type of macrophage might be able to exert a myocardial protective effect in AMI. Nevertheless, the dataset did not specify any specific types of macrophages. Therefore, further studies on the types of macrophages and their involvement in AMI could be a direction worthy of future investigation.

Although our study had a relatively large sample size, it is crucial to recognize the limitations of our approach. Firstly, as this was a retrospective analysis, there may been inherent biases in the data collection and analysis. Secondly, some profiles used in our analysis were from peripheral blood mononuclear cells, circulating endothelial cells and monocyte, only the profiles in GSE76591 dataset were from human heart tissue, which may not accurately reflect the gene expression in heart tissue. Therefore, further studies should be conducted to validate our findings using heart tissue samples. Finally, additional studies are warranted to investigate the molecular mechanisms that govern the regulation of ferroptosis in acute myocardial infarction (AMI) and its interplay with the immune system.

## Conclusion

In conclusion, our study provides valuable insights into the molecular mechanisms involved in AMI and presents a potential diagnostic biomarker for this condition. The XIST and OIP5-AS1/miR-216a/PIK3CA axes may regulate ferroptosis in AMI, which opens up a new avenue for the development of therapeutic strategies. Based on our findings, it is suggested that immune cells may have a significant impact on the key DE-FRGs biomarker, indicating the importance of exploring the interplay between the immune system and ferroptosis in AMI. Our study establishes a strong foundation for future research endeavors focused on unraveling the molecular mechanisms that underlie acute myocardial infarction (AMI). Additionally, we propose a potential biomarker that holds promise for diagnosing this condition. More in-depth animal experiments and clinical validation will further enhance the reliability of the present results.

### Supplementary Information

**Additional file 1: Supplementary Material Figures 1-5: **The assessment results about sample distribution and data reliability for GSE59867 dataset, GSE76591 dataset, GSE97320 dataset, GSE168149 dataset and GSE66360 dataset. (A): Box Plots of GEO2R show the distribution of values for each sample in the dataset to assess the sample quality and to exclude significantly discrete samples; (B): Expression Density Plots of GEO2R are complementary to box plots. By observing whether the normalized data in the Expression Density Plot matches the normal distribution, the suitability of the data for differential expression analysis is determined, which in turn ensures the reliability of the final data analysis results; (C): Plot of sample quartiles: the points in the plot are distributed along a straight line, indicating that the values of the moderated t-statistic calculated from the sample data during testing follow their theoretical predicted distribution.

## Data Availability

In this study, we conducted an analysis of publicly available datasets, which can be accessed at https://www.ncbi.nlm.nih.gov/geo/query/acc.cgi, including GSE59867, GSE97320, GSE76591, GSE168149, GSE66360 and FerrDb (http://www.zhounan.org/ferrdb) database.
