# Supplementary material for: Integrated analysis and validation of ferroptosis-related genes and immune infiltration in acute myocardial infarction
Source: BMC Cardiovasc Disord. 2024 Feb 24;24:123. doi: 10.1186/s12872-023-03622-z (PMC10893752; doi:10.1186/s12872-023-03622-z)
Supplement: Supplementary file 1 — Additional file 1: Supplementary Material Figures 1-5: The assessment results about sample distribution and data reliability for GSE59867 dataset, GSE76591 dataset, GSE97320 dataset, GSE168149 dataset and GSE66360 dataset. (A): Box Plots of GEO2R show the distribution of values for each sample in the dataset to assess the sample quality and to exclude significantly discrete samples; (B): Expression Density Plots of GEO2R are complementary to box plots. By observing whether the normalized data in the Expression Density Plot matches the normal distribution, the suitability of the data for differential expression analysis is determined, which in turn ensures the reliability of the final data analysis results; (C): Plot of sample quartiles: the points in the plot are distributed along a straight line, indicating that the values of the moderated t-statistic calculated from the sample data during testing follow their theoretical predicted distribution. [file 12872_2023_3622_MOESM1_ESM.zip › Supplementary Material Figures.docx]

**Supplementary Material Figures**

**Supplementary Material Figures 1-5:** The assessment results about sample distribution and data reliability for GSE59867 dataset, GSE76591 dataset, GSE97320 dataset, GSE168149 dataset and GSE66360 dataset. **(A):** Box Plots of GEO2R show the distribution of values for each sample in the dataset to assess the sample quality and to exclude significantly discrete samples; **(B):** Expression Density Plots of GEO2R are complementary to box plots. By observing whether the normalized data in the Expression Density Plot matches the normal distribution, the suitability of the data for differential expression analysis is determined, which in turn ensures the reliability of the final data analysis results; **(C):** Plot of sample quartiles: the points in the plot are distributed along a straight line, indicating that the values of the moderated t-statistic calculated from the sample data during testing follow their theoretical predicted distribution.
